# *Elsholtzia ciliata* (Thunb.) Hyland: A Review of Phytochemistry and Pharmacology

**DOI:** 10.3390/molecules27196411

**Published:** 2022-09-28

**Authors:** Fulin Wang, Xue Liu, Yueru Chen, Ying An, Wei Zhao, Lu Wang, Jinli Tian, Degang Kong, Yang Xu, Yahui Ba, Honglei Zhou

**Affiliations:** College of Pharmacy, Shandong University of Traditional Chinese Medicine, Jinan 250355, China

**Keywords:** *Elsholtzia ciliata* (Thunb.) Hyland, phytochemistry composition, pharmacological activities

## Abstract

In this paper, the confusion of the sources of medicinal materials was briefly expounded, and the differences among the varieties were pointed out. At the same time, the chemical components and pharmacological properties of *Elsholtzia ciliata* (Thunb.) Hyland (*E. ciliata*) were reviewed. The structures of 352 compounds that have been identified are listed. These mainly include flavonoids, terpenoids, phenylpropanoids, alkaloids, and other chemical components. They have antioxidant, anti-inflammatory, antimicrobial, insecticidal, antiviral, hypolipidemic, hypoglycemic, analgesic, antiarrhythmic, antitumor, antiacetylcholinesterase, and immunoregulator activities. At present, there are many researches using essential oil and alcohol extract, and the researches on antioxidant, anti-inflammatory, anti-microbial, and other pharmacological activities are relatively mature. This paper aims to summarize the existing research, update the research progress regarding the phytochemicals and pharmacology of *E. ciliate*, and to provide convenience for subsequent research.

## 1. Introduction

*Elsholtzia ciliata* (Thunb.) Hyland belongs to the genus *Elsholtzia*, family Lamiaceae. In the clinical application of traditional Chinese medicine, the aerial parts of *Mosla chinensis* Maxim (MCM) and *Mosla chinensis* Maxim cv. Jiangxiangru (JXR) are used as *E. ciliata*. MCM is mostly wild, and JXR is the cultivated product of MCM, which was often confused with *Elsholtzia splendens* Nakai ex F. Maek. before [1]. However, Ganpei Zhu believes that JXR has obvious plant morphological differences from MCM. The plant height of JXR can reach 25–66 cm. The stem has gray, white curly pubescence. The leaf blade is broadly lanceolate to lanceolate, and the leaf margin is obviously serrate. The bracts are obovate and ovate. Calyx lobes are triangular lanceolate in shape. There is a hair ring at the base of the crown tube. Nutlets are yellowish brown and nearly round, with lightly carved surface, reticulate and flattened inside. MCM plants are shorter. Stem inversely pilose. Leaf blade linear to linear lanceolate, leaf margin serrate, inconspicuous. Bracts ovate orbicular. Calyx lobes subulate. There is no hairy ring at the base of the crown. Nutlets are nearly spherical, brown, with deep carving on the surface, and uneven in the mesh [2]. Therefore, JXR should be listed as an independent variety [3].

*E. ciliata* is a herbaceous plant distributed in Russia (Siberia), Mongolia, Korea, Japan, India, the Indochina peninsula, and China, while in Europe and North America it was also introduced and cultivated. In China, it is produced almost all over the country, except Xinjiang and Qinghai. It has low requirements for growth environment, a short growth cycle, flowering period from July to October, and harvest in summer and autumn [4,5,6]. 

Traditional Chinese medicine theory believes that *E. ciliata* has a spicy flavour and a lukewarm nature. It also has the effect of inducing diaphoresis and relieving superficies, removing dampness for regulating the stomach and inducing diuresis for removing edema. The following is a review of chemical compositions and pharmacological activities.

## 2. Chemical Constituents

A total of **352** compounds have been identified from *E. ciliata*. Among the chemical components of *E. ciliata*, flavonoids and terpenoids are the main components, which make *E. ciliata* have more obvious antimicrobial, anti-inflammatory, and antioxidant effects. Terpenoids such as 3-carene and some aromatic compounds such as carvacrol exhibit antimicrobial activity. Some polysaccharides can inhibit the proliferation of tumor cells, and show positive effects in immunoregulation.

**Figure 1:**
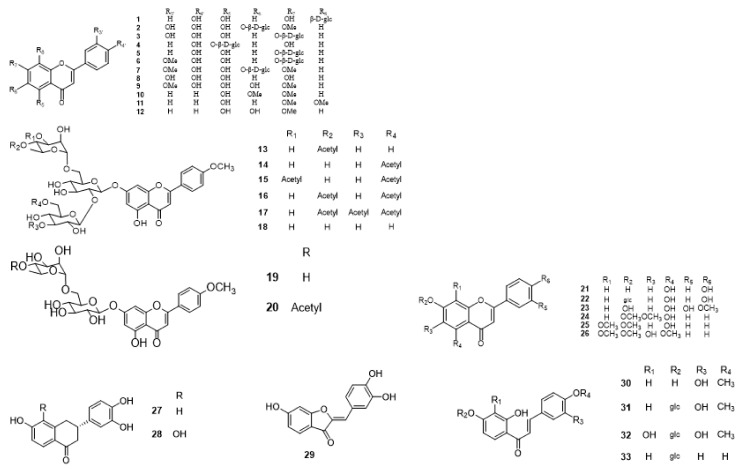

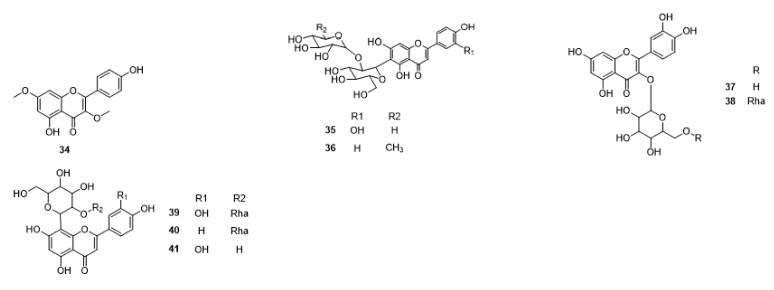

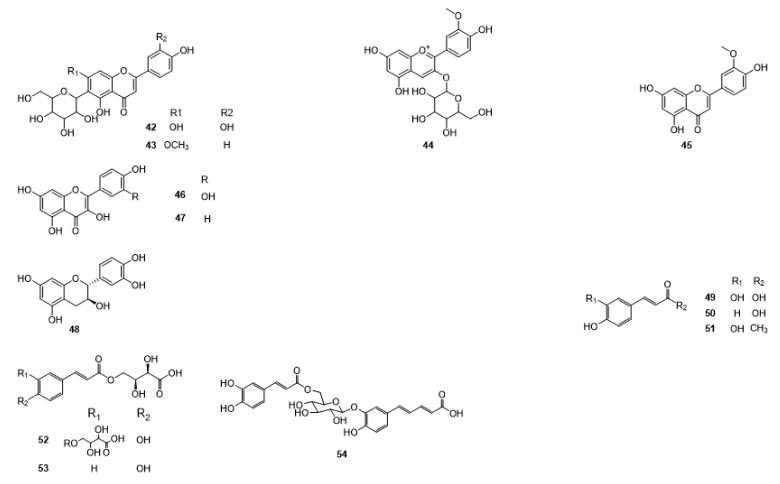

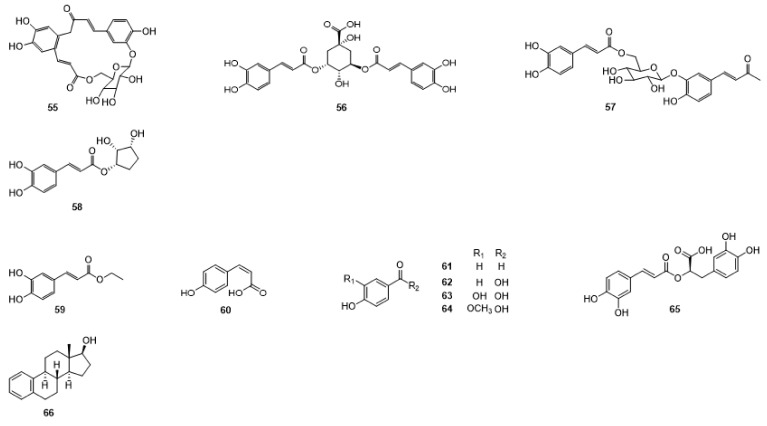

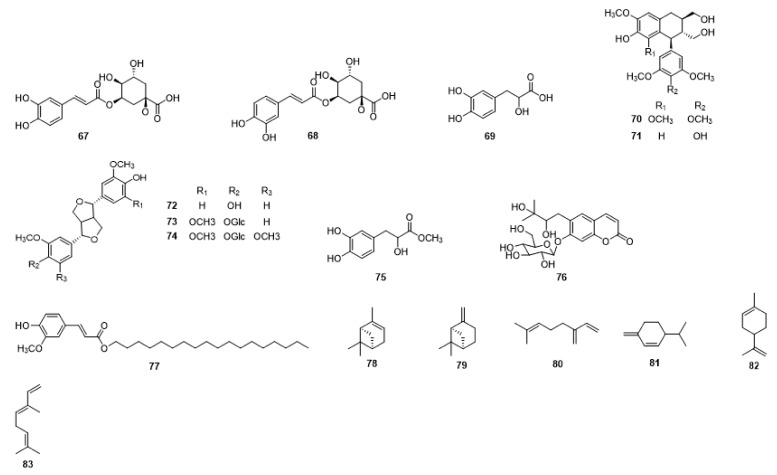

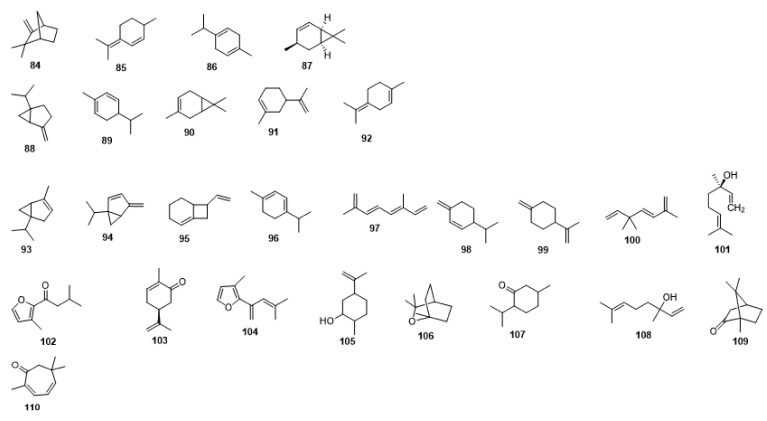

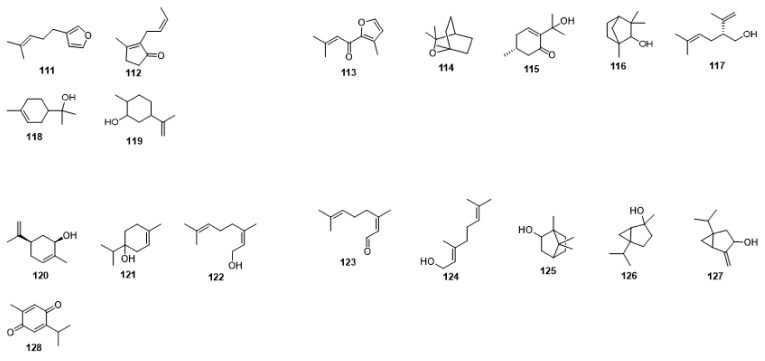

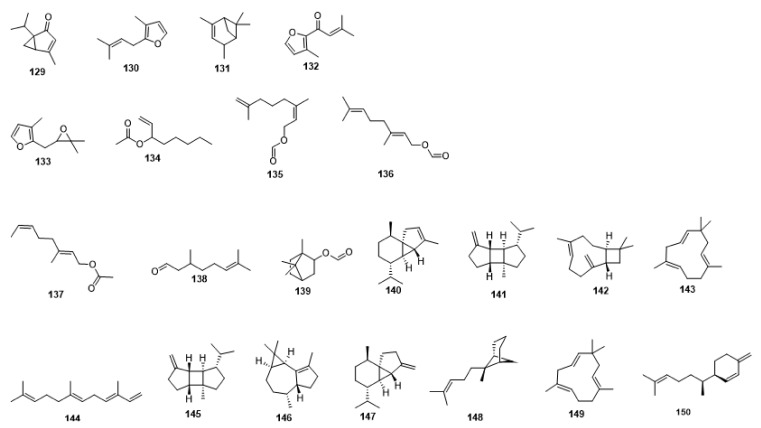

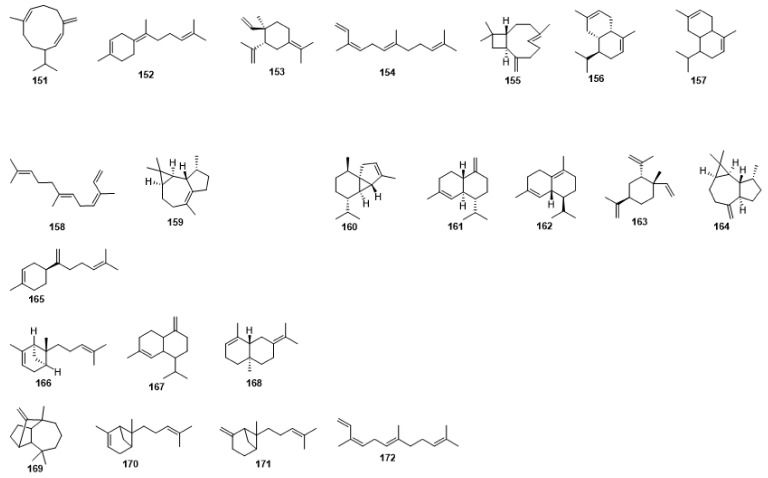

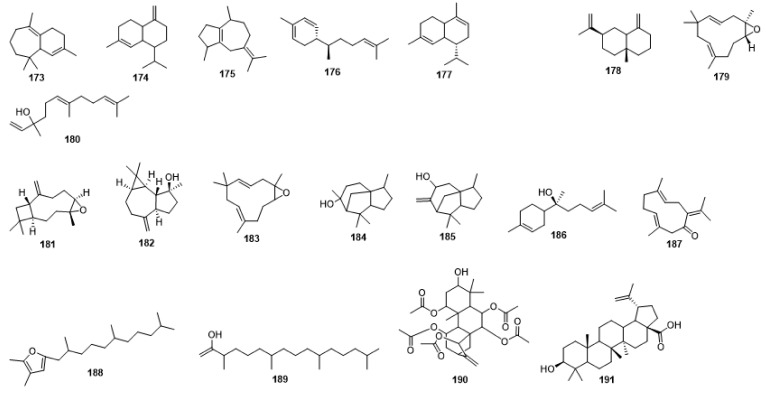

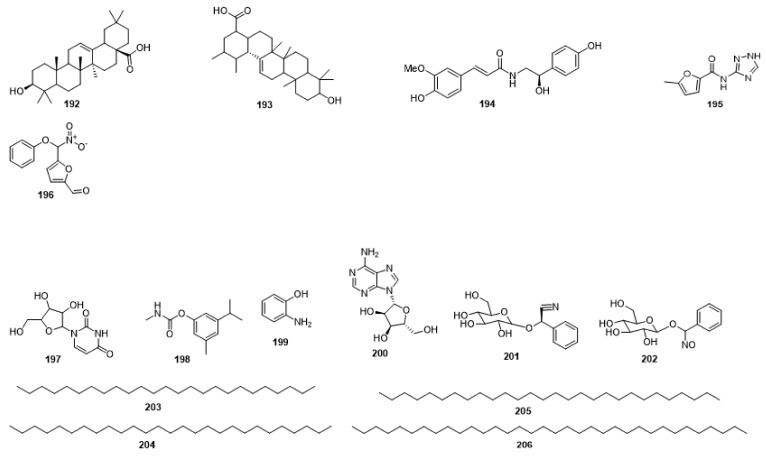

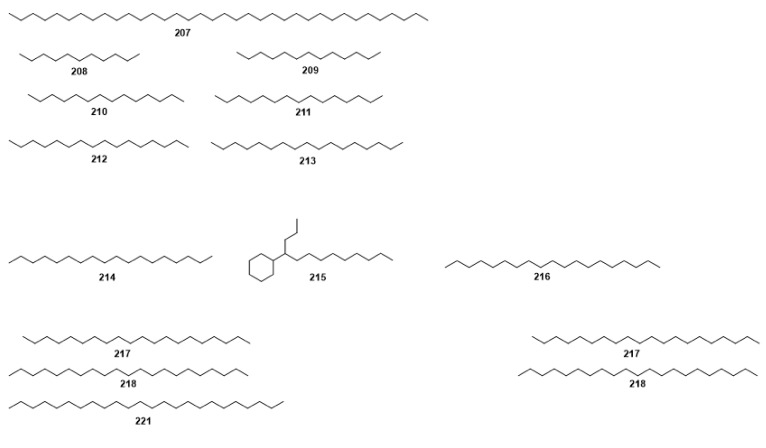

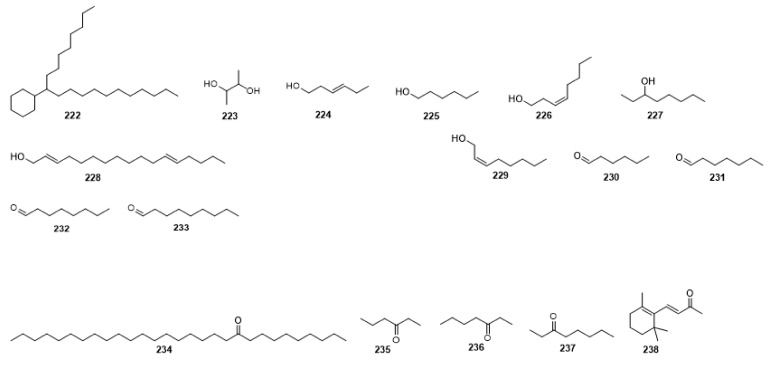

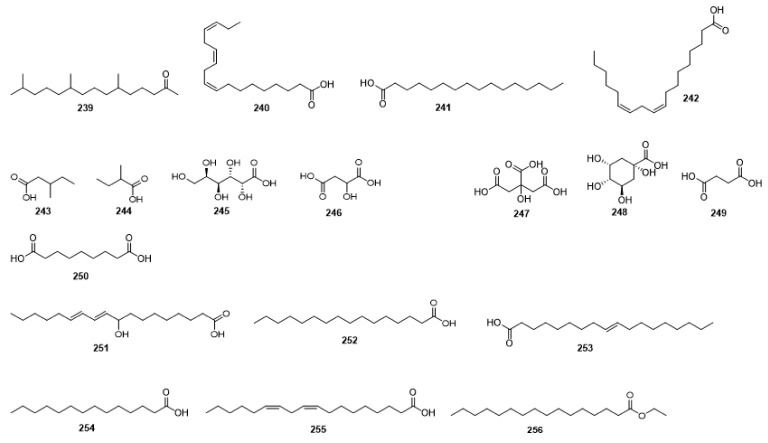

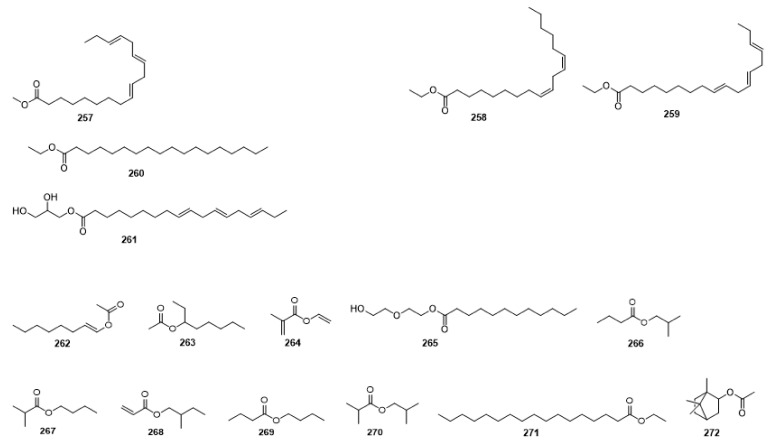

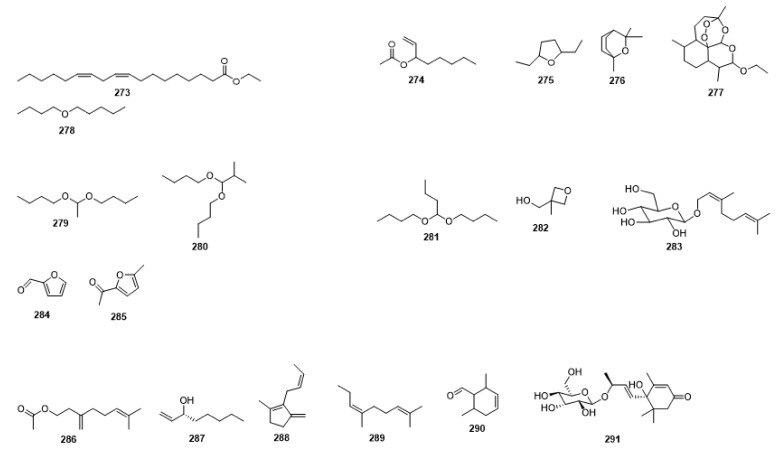

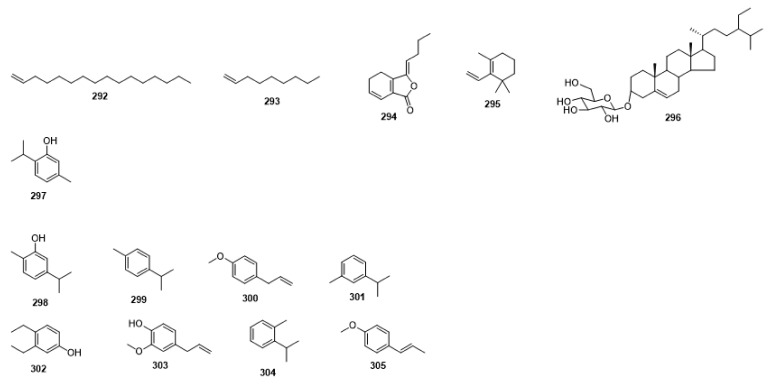

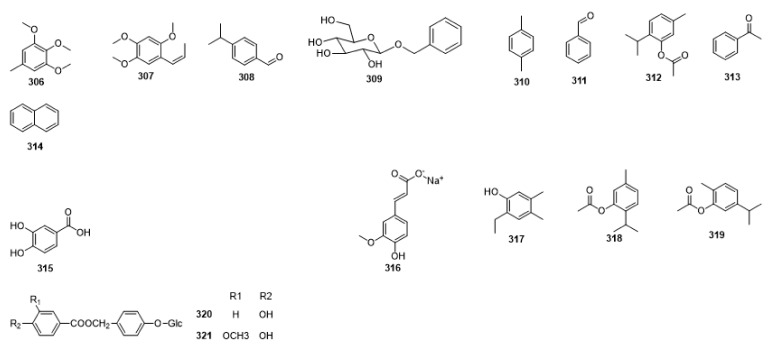

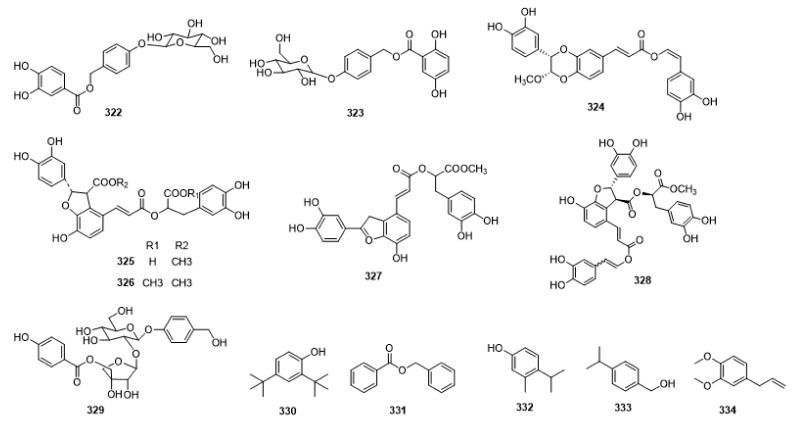

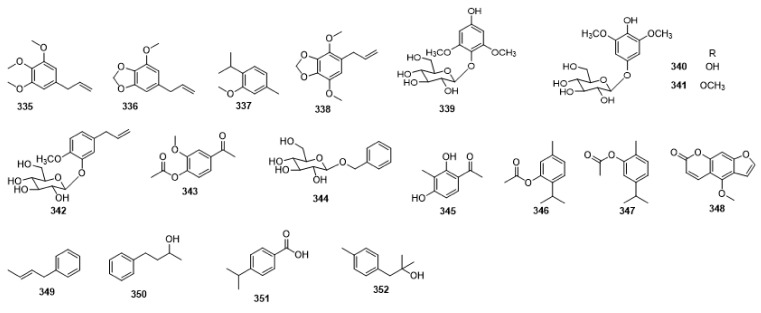
Chemical structures isolated from *E. ciliata*.

Compounds **1**–**48** are flavonoids, **49**–**77** are phenylpropanoids, **78**–**193** are terpenoids, **194**–**202** are alkaloids compounds, and **203**–**352** are other compounds. Compounds **1**–**352** are listed in Table 1 and the structures **1**–**352** are listed in Figure 1. 

## 3. Pharmacological Activities

In the traditional application of Chinese medicine, *E. ciliata* is mainly used for the treatment of summer cold, cold aversion and fever, headache without sweat, abdominal pain, vomiting and diarrhea, edema, and poor urination. Modern pharmacological studies show that *E. ciliata* has antioxidant, anti-inflammatory, antimicrobial, insecticidal, antiviral, hypolipidemic, hypoglycemic, analgesic, antiarrhythmic, antitumor, antiacetylcholinesterase, and immunoregulator activities. 

### 3.1. Antioxidant Activity

Oxidative stress refers to a state of imbalance between oxidation and antioxidant effects in vivo. It is a negative effect caused by free radicals in the body and is considered to be an important factor in aging and disease. It was reported that the essential oil of *E. ciliata* could increase catalase (CAT) activity in brain of mice by 26.94%, which may be related to the decomposition of hydrogen peroxide by CAT to reduce oxidative stress [7]. There is a phenolic substance osmundacetone in *E. ciliata* ethanol extract. In DPPH experiment, the IC_50_ value of osmundacetone was 7.88 ± 0.02 µM, indicating a certain antioxidant capacity. The inhibitory effect of osmundacetone on glutamate-induced oxidative stress in HT22 cells was studied by reactive oxygen species (ROS) method. The results showed that osmundacetone significantly reduced the accumulation of ROS and could be used as a potential antioxidant [8]. By studying the effect of *E. ciliata* methanol extract on J774A.1 murine macrophage, the evaluation of antioxidant activity showed that all the tested compounds had significant effects on ROS release under oxidative stress at the highest concentration (10 M), especially luteolin-7-*O*-*β*-D-glucopyranoside, luteolin, and 5,6,4’-trihydroxy-7,3’-dimethoxyflavone [9]. 

Various scholars studied different polarity extracts of *E. ciliata*. According to the free radical scavenging experiment of Huynh Xuan Phong, the result showed that *E. ciliata* extract had certain scavenging ability against 2,2-diphenyl-1-picrylhydrazyl (DPPH) and 2,2’-azino-bis (3-ethylbenzothiazoline -6-sulfonic acid) (ABTS), with IC50 values of 495.80 ± 17.16 and 73. 59 ± 3.18 mg/mL. [10]. In DPPH experiment, the EC_50_ values of dichloromethane extract, crude ethanol extract and n-hexane extract were 0.041 µg/µg, 0.15 µg/µg and 0.46 µg/µg, respectively, showing strong antioxidant activity. Such antioxidant capacity may be related to non-polar flavonoids and phenols contained in *E. ciliata*, among which the total phenol content of dichloromethane is 96.68 ± 0.0010 µg GAEs/mg Extract, and the total flavonoid content is 71.5 ± 0.0089 µg QEs/mg Extract. Therefore, it has the strongest antioxidant capacity [11]. Jing-en Li et al. extracted JXR ethanol extract with petroleum ether, ethyl acetate and water-saturated n-butanol, respectively, and studied the antioxidant activities of the three parts and water phase. The results indicated that ethyl acetate showed good antioxidant activities in ferric reducing antioxidant power (FRAP), DPPH, and *β*-carotene assay, which may be related to the higher flavonoid content in this extract [12]. The antioxidant ability of mytilus polysaccharide-I (MP-I) contained in JXR water extract was concentration-dependent. When the concentration was 16 mg/mL, the chelation rate of MP-I and Fe^2+^ was 87.80%. When the concentration was 20 mg/mL, the scavenging rate of DPPH free radical was 81.32%. The scavenging rate of hydroxyl radical was 81.94% [13]. The DPPH test IC_50_ of MCM essential oil and methanol extract were 1230.4 ± 12.5 and 1482.5 ± 10.9 μg/mL, respectively. Reducing power test EC_50_ were 105.1 ± 0.9 and 313.5 ± 2.5 μg/mL, respectively. *β*-Carotene bleaching assay EC_50_ were 588.2 ± 4.2 and 789.4 ± 1.3 μg/ml, respectively. The total phenolic content of the essential oil was about 1.7 times that of methanol extract, which further verified the stronger antioxidant capacity of the essential oil [14]. 

Different parts of *E. ciliata* have different antioxidant capacity. Lauryna Pudziuvelyte et al. used DPPH, ABTS, FRAP, and cupric ion reducing antioxidant capacity (CUPRAC) to evaluate the antioxidant activity of different parts of *E. ciliata* DPPH and ABTS results showed that total phenolics content (TPC) and total flavonoids content (TFC) amounts of ethanol extracts from *E. ciliata* flower, leaf and whole plant were the highest and had the strongest antioxidant activity. The results of FRAP and CUPRAC test showed that the ethanol extract of *E. ciliata* flower had the highest antioxidant activity. Among different parts of the ethanol extract, the content of quercetin glycosides, phenolic acids, TPC, and TFC in stem extract was the lowest, and the antioxidant activity was the lowest [4]. The ethyl acetate fraction of *E. ciliata* was purified by macroporous resin with 80% ethanol to obtain fraction E. In the DPPH experiment, the EC_50_ value of fraction E was 0.09 mg/mL, which showed the strongest antioxidant and free radical scavenging ability. The EC_50_ value of fraction E was higher than positive control butylated hydroxytoluene (0.45), butylated hydroxyanisole (0.21), and vitamin C (0.41). Hence, it can be seen that *E. ciliata* has the potential to prevent cardiovascular diseases, cancer, and other diseases caused by excess free radicals [15]. 

### 3.2. Anti-Inflammatory Activity

Compounds pedalin, luteolin-7-*O*-*β*-D-glucopyranoside, 5-hydroxy-6,7-dimethoxyflavone, and *α*-linolenic acid in the essential oil of *E. ciliata* were investigated under a lipopolysaccharide (LPS)-induced inflammatory reaction. It can inhibit ROS release, but its mechanism deserves further study [9]. LPS-induced inflammation was evaluated by the number of inflammatory mediators, i.e., tumor necrosis factor-*α* (TNF-*α*), interleukin (IL)-6, and prostaglandin E2 (PGE2). *E. ciliata* ethanol extract could significantly inhibit the secretion of inflammatory mediators, where TNF-*α* and IL-6 factors could be effectively inhibited in the stem and flower part, and the PGE2 pathway could be inhibited in the leaf part [4]. The effect of *E. ciliata* on inflammation can be further verified by studying pyretic rats caused by LPS and mononuclear macrophage RAW264.7 induced by LPS. *E. ciliata* essential oil and water decoction can reduce the contents of PGE2, TNF-*α* and other inflammatory factors to different degrees, and can reduce the content of nitric oxide (NO) in serum [16]. Excessive NO can induce the production of pro-inflammatory factors, such as TGF-*α* and IL-1*β*, and aggravate the inflammatory response [17]. JXR alleviates dextran sulfate sodium induced intestinal knot inflammation in mice by affecting the release of NO, PGE2 and other inflammatory mediators and cytokines [18]. Carvacrol in MCM can inhibit the expression of pro-inflammatory cytokines interferon-*γ* (IFN-*γ*), IL-6, and IL-17 and up-regulate the expression of anti-inflammatory factors TGF-*β*, IL-4, and IL-10, thus reducing the level of inflammatory factors, reducing the damage to cells, and achieving anti-inflammatory effects [19]. 

In the formalin-induced licking response test, the licking time of *E. ciliata* crude ethanol extract and dichloromethane extract is shortened at the late phase under 100 mg/kg dose, and the licking time of n-hexane extract is shortened at the early phase under 100 mg/kg dose, which may be related to its anti-inflammatory effect [11]. Water extract of *E. ciliata* has anti-allergic inflammatory activity and may be related to the inhibition of calcium, P38 mitogen-activated protein kinase, and nuclear factor-*κ*B expression in the human mast cell line [20]. 

### 3.3. Antimicrobial Activity

Different polar extracts of *E. ciliata* demonstrated significant differences in inhibition ability with regard to microorganism. The results showed that dichloromethane fraction had the strongest inhibitory activity on *Candida albicans* with minimum inhibitory concentration (MIC) of 62.5 µg/mL, while n-hexane fraction had the strongest inhibitory effect on *Escherichia coli* with MIC of 250 µg/mL [11]. The ethyl acetate extract of JXR had strong inhibitory effect on *Rhizopus oryzae*, with the inhibition zone diameter of 13.7 ± 2.7 mm, MIC of 5 mg/mL and minimum bactericidal concentration (MBC) of 5 mg/mL [12]. The MIC of JXR petroleum ether extract, n-butanol extract and ethanol extract against *Escherichia coli*, *Staphylococcus aureus* and *Bacillus subtilis* were 31.25 μg/mL, and the MIC of ethyl acetate extract was 15.60 μg/mL [21]. The carbon dioxide extract of *E. ciliata* demonstrated a certain inhibitory effect on *Staphylococcus aureus*, *Salmonella paratyphoid,* and other microorganisms. When the concentration of the extract was 0.10 g/mL, the inhibitory effect on *Staphylococcus aureus* was the most obvious, and the diameter of the inhibition zone is 19.7 ± 0.1 mm [22].

According to existing research reports, *E. ciliata* is rich in essential oil, which contains abundant antibacterial ingredients and can inhibit a variety of microorganisms, so it has research significance and value. The main antibacterial active components of the essential oil of *E. ciliata* are thymol, carvacrol, and p-Cymene, which have inhibitory effect on *Staphylococcus aureus*, *Methicillin-resistant Staphylococcus aureus* and *Escherichia coli*. MIC were 0.39 mg/mL, 3.12 mg/mL and 1.56 mg/mL, and the diameters of inhibition zone were 21.9 ± 0.1230, 18.2 ± 0.0560, and 16.7 ± 0.0115 nm, respectively [23]. The essential oil in *E. ciliata* flowers, stems, and leaves had inhibitory effects on *Escherichia coli*, *Staphylococcus aureus*, *Salmonella typhi*, *Klebsiella pneumoniae,* and *Pseudomonas aeruginosa.* Both of them had the strongest inhibitory effect on *Staphylococcus aureus* with the inhibitory zone diameter of 12.2 ± 0.4 and 11.2 ± 0.1 mm, respectively [24]. Other relevant findings suggest that JXR essential oil may affect the formation of *Staphylococcus aureus* biofilm, so as to achieve bacteriostatic effect on its growth. The MIC of JXR essential oil to *Staphylococcus aureus* was 0.250 mg/mL. When the concentration was 4MIC, the inhibition rate of essential oil to *Staphylococcus aureus* biofilm formation could reach 91.3%, and the biofilm clearance rate was 78.5%. The MIC of carvacrol, thymol, and carvacrol acetate against *Staphylococcus aureus* were 0.122, 0.245, and 0.195 mg/mL, respectively, which were the effective antibacterial components of essential oil. Carvacrol, carvacryl acetate, *α*-cardene, and 3-carene had strong inhibitory effects on the formation of *Staphylococcus aureus* biofilm, and the inhibition rates were more than 80% at 1/4 MIC (0.0305, 1.4580, 0.1267 and 2.5975 mg/mL, respectively) [25,26]. In another study, Li Cao et al. studied the inhibitory effect of MCM essential oil on 17 kinds of microorganisms, among which, It significantly inhibited *Chaetomium globosum, Aspergillus fumigatus* and *Candida rugosa*. The antibacterial zone diameters were 16.3 ± 0.58, 15.0 ± 1.00, 16.0 ± 0.00 mm, and MIC were 31.3, 62.5, 62.5 μg/mL, respectively [14]. It also has obvious inhibitory effect on *Bacillus subtilis* and *Salmonella enteritidis*, which might be related to the terpenes contained, but this opinion remains to be verified [27]. Thymol and carvacrol are the main antibacterial components of MCM. Caryophyllene oxide can be used in the treatment of dermatomycosis, especially in the short-term treatment of mycosis ungualis [28]. The bactericidal mechanism of essential oil may be due to the fact that active components such as carvacrol can damage cell membranes and alter their permeability [29].

The extract of MCM had a significant inhibitory effect on the spore germination of *Aspergillus flavus* and could significantly change the morphology of *Aspergillus flavus* mycelia, podocytes, and sporophytes, with a MIC of 0.15 mg/mL [30]. The germination rate of *Penicillium digitorum* treated with carvacrol significantly decreased, the mechanism may be that carvacrol can change the surface morphology of mycelia, and the cavity rate of mycelia increased with the increase of carvacrol concentration. The permeability of the cell membrane of bacteria increases, causing an electrolyte imbalance in bacteria. As a result, the sugar content and nutrients in bacteria are reduced, so as to achieve bacteriostasis. The MIC and MBC of carvacrol against *Penicillium digitorum* were 0.125 and 0.25 mg/mL, respectively [31]. 

### 3.4. Insecticidal Activity

Some studies have shown that *E. ciliata* has an insecticidal effect. The repellency rate of *E. ciliata* essential oil to *Blattella germanica* was 64.50%, no significant difference from positive control diethyltoluamide (DEET) (*p* < 0.05). RD_50_ of *E. ciliata* essential oil was 218.634 µg/cm^2^, which was better than DEET (650.403 µg/cm^2^) [32]. Contact toxicity IC_50_ of *E. ciliata* essential oil to *Liposcelis bostrychophila* was 145.5 μg/cm^2^, and fumigation toxicity IC_50_ was 475.2 mg/L. (R)-carvone. Dehydroelsholtzia ketone and elsholtzia ketone are the active components of *E. ciliata* essential oil against *Liposcelis bostrychophila*. The IC_50_ of contact toxicity were 57.0, 151.5, and 194.1 μg/cm^2^, and those of fumigantion toxicity were 417.4, 658.2, and 547.3 mg/L, respectively [6]. Carvone and limonene are the two main components in *E. ciliata* essential oil. The ability of *E. ciliata* essential oil, carvone, and limonene against *Tribolium castaneum* larvae and adults was evaluated by a contact toxicity test and fumigation assay. Contact toxicity test showed that the LD_50_ of *E. ciliata* essential oil, carvone, and limonene to *Tribolium castaneum* adults were 7.79, 5.08, and 38.57 mg/Adult, respectively, and 24.87, 33.03, and 49.68 mg/Larva to *Tribolium castaneum* larvae. The results of fumigation toxicity test showed that LC_50_ of *Tribolium castaneum* adults were 11.61, 4.34, and 5.52 mg/L Air, respectively, and LC_50_ of *Tribolium Castaneum* larvae were 8.73, 28.71, and 20.64 mg/L Air, respectively [5]. Thymol, carvacrol, and *β* -thymol contained in JXR essential oil had significant fumigation toxicity against *Mythimna Separate, Myzus Persicae, Sitophilus Zeamais, Musca domestica,* and *Tetranychus cinnabarinus*, among which *β*-thymol has the strongest activity. The IC_50_ values for the five pests were 10.56 (9.26–12.73). 14.13 (11.84–16.59), 88.22 (78.53–99.18), 10.05 (8.63–11.46), and 7.53 (6.53–8.79) μL/L air, respectively [33]. Determined by the immersion method, the LC_50_ of MCM essential oil against *Aedes albopictus* larvae and pupae at four instars were 78.820 and 122.656 μg/mL, respectively. The chemotaxis activity of MCM essential oil was evaluated by the method of effective time of human local skin coating. When the dose was 1.5 mg/cm^2^, the complete protection time of *Aedes albopictus* was 2.330 ± 0.167 h [34]. From this point of view, *E. ciliata* essential oil has the development potential as a natural anti-insect agent. It provides a basis for the development and utilization of pesticide dosage forms.

*Leishmania mexicana* can cause cutaneous leishmaniasis. *E. ciliata* essential oil had anti-leishmania activity with IC_50_ of 8.49 ± 0.32 nL/mL. *Leishmania mexicana mexicana* was treated with a survival rate of 0.38 ± 0.00 %. Selectivity indices were 5.58 and 1.56 for mammalian cell WI38 and J774, respectively. This provides a reference for the treatment of cutaneous leishmaniasis [35]. *E. ciliata* water extract has an obvious anti-*trichomonas vaginalis* effect, i.e., can destroy the insect body structure, to achieve the purpose of killing insects. The results of in vitro experiments showed that the lowest effective concentration of *E. ciliata* water extract was 62.5 mg/mL, and the lowest effective time was 12 h. When the concentration was 250 mg/mL, all *Trichomonas vaginalis* could be killed for 4 h. This experiment provides a new idea for the clinical treatment of vaginal trichomoniasis [36]. 

### 3.5. Antiviral Activity

T helper 17 (Th17) cells play an important role in maintaining adaptive immune balance, and an excess of Th17 cells can cause inflammation. Carvacrol plays an anti-influenza virus role by reducing the proportion of Th17 cells significantly increased by influenza virus A infection. It can be used as a potential antiviral drug and can also be used to control inflammation caused by influenza virus A infection [19]. Mice with viral pneumonia modeled by A/PR/8/34 (H1N1) virus were treated with low, medium and high dose of MCM total flavonoids. Lung index of the three dose groups were 12.81 ± 3.80, 11.65 ± 2.58, 11.45 ± 2.40 mg/g, respectively, compared with the infection group 16.05 ± 3.87 mg/g, the inhibition rates were 20.18%, 27.41%, 28.66%, respectively [37]. *E. ciliata* ethanol extract has an inhibitory effect on the proliferation of avian infectious bronchitis virus, which may be related to the increased expression of three antiviral genes suppressor of cytokine signaling 3 (SOCS3), 2′-5′-oligoadenylate synthetase-like (OASL), and signal transducer and activator of transcription 1 (STAT1) in H1299 cells treated with extract, and this inhibitory effect shows a certain concentration dependence. In addition, the extract had no cytotoxicity when the concentration was less than 0.3 g/mL [38]. Above experiments provide new possibilities for the treatment of inflammation caused by the virus.

A/WSN/33/2009 (H1N1) virus was used to infect Madin-Darby canine kidney cells to explore the antiviral activity of phenolic acids from MCM in vitro. The survival rate of the cells treated with the compound 3-(3,4-dihydroxyphenyl) acrylic acid 1-(3,4-dihydroxyphenyl)-2-methoxycarbonylethyl and methyl lithospermate were higher than 80%, and the inhibition rate of virus at 100 μmol/L were 89.28% and 98.61%, respectively [39]. In another study, the lung index of low, medium, and high dose of MCM water extract on mice infected by A/PR8 influenza virus was 1.21 ± 0.22%, 1.12 ± 0.17%, and 0.94 ± 0.21%, respectively. Compared to the virus-infected group 1.80 ± 0.29 %, the inhibition rates were 32.78%, 37.78% and 47.78%, respectively. The extracts of the three groups can increase the amounts of IL-2 and IFN-*γ* in serum of mice, and promote the antiviral ability of the body indirectly or directly [40]. Fluoranthene is a compound with antiviral activity extracted from *E. ciliata*. It has a certain inhibitory effect on two enveloped viruses, sindbis virus, and murine cytomegalovirus, with the lowest effective concentrations of 0.01 and 1.0 μg/mL, respectively. However, its biological effects are complex, and its clinical safety and effectiveness need further research [41].

### 3.6. Hypolipidemic Activity

The hypolipidemic activity of *E. ciliata* ethanol extract was evaluated by determining the effects on the contents of triglyceride and total cholesterol in serum of mice in vivo and the proliferation of 3T3-L1 preadipocytes in vitro. The results showed that the levels of triglyceride and total cholesterol in serum of mice treated with the extract were decreased, and the differentiation and accumulation of 3T3-L1 preadipocytes were also effectively inhibited. The levels of genes associated with adipogenesis, such as peroxisome proliferator activated receptor *γ* (PPAR*γ*), fatty acid synthase (FAS), and adipocyte fatty acid-binding protein 2 (aP2) were also significantly reduced. In addition, serum leptin content in *E. ciliata* ethanol extract treatment group was lower than that in obese mice, which may be due to the reduction of fat content. By this token, the action mechanism of *E. ciliata* lowering blood lipids may be to inhibit the expression of genes related to fat cell formation. However, the specific mechanism needs further study [42]. 

### 3.7. Antitumor Activity

Pudziuvelyte, L. et al. extracted essential oil from *E. ciliata* fresh herbs, lyophilized herbs, and dried herbs, respectively. In in vitro experiments, three kinds of essential oil presented significant inhibition of proliferation effect on the human glioblastoma (U87), pancreatic cancer (PANC-1), and triple negative breast cancer (MDA-MB231) cells, with EC_50_ values ranging from 0.017% to 0.021%. However, *E. ciliata* ethanol extract did not show cytotoxicity in this experiment [43]. The antitumor activity of origin processing integration technology and traditional cutting processing technology of *E. ciliata* was evaluated by measuring the effect of the decoction and essential oil on the average optical density of TNF-*α* in rat lung tissue. Average optical densities of water decocted solution and essential oil of traditional cutting *E. ciliata* were 0.530 ± 0.071 and 0.412 ± 0.038, respectively, and those of integration processing technology of origin were 0.459 ± 0.051 and 0.459 ± 0.051, respectively. Compared with the blank group (0.299 ± 0.028), there were varying degrees of increase [44]. In vitro experiments of JXR pectin polysaccharide (MP-A40) showed that the proliferation of human leukemic cell line K562 was affected by MP-A40. When the concentration of MP-A40 was 500μg/mL, the inhibition rate was 31.32% [45]. 

### 3.8. Immunoregulatory Activity

Macrophages can regulate apoptosis by producing NO and other effecting molecules. Macrophage RAW 264.7 cells treated by JXR pectin polysaccharide (MP-A40) showed an obvious increase in NO production. Moreover, it’s concentration-dependent. When the concentration of MP-A40 was as low as 10 μg/mL, NO production was still 15 times that of negative control [45]. Mice treated with cyclophosphamide had elevated levels of free radicals, increasing aggression towards immune organs, and decreased thymus and spleen indices. Polysaccharide MP can scavenge free radicals and promote the proliferation of ConA-induced T cells and LPS-induced B cells. To a certain extent, the immunosuppression induced by cyclophosphamide can be alleviated [13,46]. However, the potential immunomodulatory mechanism of polysaccharide remains to be further studied.

### 3.9. Others

Different polar ethanol extracts of JXR had different degrees of inhibition on *α*-glucosidase activity. Therefore, it has certain hypoglycemic activity. When the polar ethanol extract concentration was 4.0 mg/mL, the inhibition rate of petroleum ether extract was 93.8%, IC_50_ was 0.339 mg/mL, and the inhibition rate of ethyl acetate extract was 92.8%, IC_50_ was 0.454 mg/mL. The essential oil prepared by steam distillation, petroleum ether cold extraction, and petroleum ether reflux extraction also showed significant inhibition of *α*-glucosidase at the concentration of 0.25 mg/mL, and the inhibition rates were more than 90% [47]. 

The results of the formalin-induced Licking test showed that *E. ciliata* crude ethanol extract has analgesic effect on the early stage of reaction (0–5 min) [11]. 

THE Langendorff perfused isolated rabbit heart model was used. When *E. ciliata* essential oil was added into perfusate, QRS interval was increased, QT interval was shortened, AND action potentials upstroke amplitude was decreased, and activation time was prolonged when the concentration of *E. ciliata* essential oil was increased in the range of 0.01–0.1 μL/mL, and showed concentration dependence. This may be due to the fact that sodium channel block can increase the threshold of action potential generation, prolong the effective refractory period, and inhibit the zero-phase depolarization of late depolarization. The reduction of action potential duration can reduce the occurrence of early depolarization. This experiment provides theoretical basis for *E. ciliata* in the treatment of arrhythmia [48].

7-*O*-(6-*O*-acetyl)-*β*-D-glucopyranosyl-(1→2)[(4-oacetyl)-*α*-L-rhamnopyranosyl-(1→6)]-*β*-D-glucopyranoside in methanol extract of *E. ciliata* was hydrolyzed to obtain acacetin. The IC_50_ of acacetin against acetylcholinesterase was 50.33 ± 0.87 μg/mL, which showed a significant inhibitory effect on acetylcholinesterase activity, which may hold promise for Alzheimer’s disease treatment [49].

**Table 1:** Compounds [5,6,9,11,18,22,24,26,27,28,33,34,39,43,49,50,51,52,53,54,55,56,57,58,59,60,61,62,63,64,65,66,67,68,69].

No.	Compound Name	Formula	*E. ciliata*	JXR	MCM	References
Flavonoids						
1	vitexin	C_21_H_20_O_10_	+	-	-	[11]
2	pedalin	C_22_H_22_O_12_	+	-	-	[11]
3	luteolin-7-*O*-*β*-D-glucopyranoside	C_20_H_20_O_11_	+	-	-	[11]
4	apigenin-5-*O*-*β*-D-glucopyranoside	C_20_H_20_O_10_	+	-	-	[11]
5	apigenin-7-*O*-*β*-D-glucopyranoside	C_20_H_20_O_10_	+	-	-	[11]
6	chrysoeriol-7-*O*-*β*-D-glucopyranoside	C_21_H_22_O_11_	+	-	-	[11]
7	7,3′-dimethoxyluteolin-6-*O*-*β*-D-glucopyranoside	C_21_H_24_O_12_	+	-	-	[11]
8	luteolin	C_15_H_10_O_6_	+	+	-	[11,18]
9	5,6,4′-trihydroxy-7,3′-dimethoxyflavone	C_17_H_14_O_7_	+	-	-	[9]
10	5-hydroxy-6,7-dimethoxyflavone	C_17_H_14_O_5_	+	-	-	[9]
11	5-hydroxy-7,8-dimethoxyflavone	C_17_H_14_O_5_	+	-	-	[9]
12	negletein	C_16_H_12_O_5_	-	-	+	[50]
13	acacetin-7-*O*-[*β*-D-glucopyranosyl(1″″→2″)-4‴-*O*-acetyl-*α*-L-rhamnopyranosyl(1‴→6″)]-*β*-D-glucopyranoside	C_36_H_44_O_20_	+	-	-	[51]
14	acacetin-7-*O*-[6″″-*O*-acetyl-*β-*D-glucopyranosyl(1″″→2″)-*α*-L-rhamnopyranosyl(1‴→6″)]-*β-*D-glucopyranoside	C_36_H_44_O_20_	+	-	-	[51]
15	acacetin-7-*O*-[6″″-*O*-acetyl-*β*-D-glucopyranosyl(1″″→2″)-3‴-*O*-acetyl-*α*-L-rhamnopyranosyl(1‴→6″)]-*β*-D-glucopyranoside	C_38_H_46_O_21_	+	-	-	[51]
16	acacetin-7-*O*-[6″″-*O*-acetyl-*β*-D-glucopyranosyl(1″″→2″)-4‴-*O*-acetyl-*α*-L-rhamnopyranosyl(1‴→6″)]-*β*-D-glucopyranoside	C_38_H_46_O_21_	+	-	-	[51]
17	acacetin-7-*O*-[3″″,6″″-di-Oacetyl-*β*-D-glucopyranosyl(1″″→2″)-4‴-*O*-acetyl-*α*-L-rhamnopyranosyl(1‴→6″)]-*β*-D-glucopyranoside	C_40_H_45_O_25_	+	-	-	[51]
18	7-*O*-*β*-D-glucopyranosyl-(1 → 2)[*α*-L-rhamnopyranosyl(1 → 6)]-*β*-D-glucopyranoside	C_34_H_42_O_19_	+	-	-	[49]
19	linarin	C_28_H_32_O_14_	+	-	-	[51]
20	acacetin-7-*O*-[4‴-*O*-acetyl-*α*-L-rhamnopyranosyl(1‴→6″)]-*β*-D-glucopyranoside	C_30_H_34_O_15_	+	-	-	[51]
21	apigenin	C_15_H_10_O_5_	+	-	+	[50,51]
22	apigetrin	C_21_H_20_O_10_	+	-	-	[51]
23	diosmetin	C_16_H_12_O_6_	+	-	-	[51]
24	5-hydroxy-6,7-dimethoxyflavone	C_17_H_14_O_5_	+	-	-	[51]
25	5-hydroxy-7,8-dimethoxyflavone	C_17_H_14_O_5_	+	-	-	[51]
26	6-hydroxy-5,7,8-trimethoxyflavone	C_18_H_16_O_6_	+	-	-	[51]
27	butin	C_16_H_14_O_4_	+	-	-	[51]
28	isookanin	C_16_H_14_O_5_	+	-	-	[51]
29	sulfuretin	C_15_H_10_O_5_	+	-	-	[51]
30	3,2′,4′-trihy-droxy-4-methoxychalcone	C_16_H_14_O_5_	+	-	-	[51]
31	3,2′,4′-trihy4′-*O*-*β*-D-glucopyranosyl-3,2′-dihydroxy-4-methoxychalcone	C_21_H_24_O_10_	+	-	-	[51]
32	okanin-4-methoxy-4′-*O*-*β*-D-glucopyranoside	C_21_H_24_O_11_	+	-	-	[51]
33	neoisoliquiritin	C_20_H_22_O_9_	+	-	-	[51]
34	kumatakenin	C_17_H_14_O_6_	+	-	-	[52]
35	isoorientin-2′′-*O*-rhamnoside	C_27_H_30_O_15_	-	+	-	[18]
36	isovitexin-2′′-*O*-rhamnoside	C_27_H_30_O_14_	-	+	-	[18]
37	quercetin-3-*O*-rutinoside	C_27_H_30_O_16_	-	+	-	[18]
38	quercetin-3-*O*-glucoside	C_21_H_20_O_12_	-	+	-	[18]
39	orientin-2′′-*O*-rhamnoside	C_27_H_30_O_15_	-	+	-	[18]
40	vitexin-2′′-*O*-rhamnoside	C_27_H_30_O_14_	-	+	-	[18]
41	orientin	C_21_H_20_O_11_	-	+	-	[18]
42	isoorientin	C_21_H_20_O_11_	-	+	-	[18]
43	swertisin	C_22_H_22_O_10_	-	+	-	[18]
44	peonidin-3-*O*-glucoside	C_22_H_22_O_11_	-	+	-	[18]
45	chrysoeriol	C_16_H_12_O_6_	-	+	-	[50]
46	quercetin	C_15_H_10_O_7_	-	+	-	[50]
47	kaempferol	C_15_H_10_O_6_	-	+	-	[53]
48	catechin	C_15_H_14_O_6_	+	-	-	[54]
Phenylpropanoids						
49	caffeic acid	C_9_H_8_O_4_	+	+	-	[9,18]
50	(E)-p-coumaric acid	C_9_H_8_O_3_	+	-	-	[9]
51	osmundacetone	C_10_H_10_O_3_	+	-	-	[9]
52	4-(E)-caffeoyl-L-threonic acid	C_17_H_16_O_12_	+	-	-	[9]
53	4-*O*-(E)-p-coumaroyl-L-threonic acid	C_13_H_14_O_7_	+	-	-	[9]
54	(7E,9E)-3-hydroxyavenalumic acid-3-*O*-[6′-*O*-(E)-caffeoyl]-*β*-D-glucopyranoside	C_26_H_26_O_12_	+	-	-	[51]
55	gnaphaliin C	C_25_H_24_O_11_	+	-	-	[51]
56	3,5-di-*O*-caffeoylquinic acid	C_25_H_24_O_12_	+	-	-	[51]
57	everlastoside L	C_25_H_26_O_11_	+	-	-	[51]
58	1-(3′,4′-dihydroxycinnamoyl)cyclopentane2,3-diol	C_14_H_16_O_6_	+	-	-	[51]
59	ethyl caffeate	C_11_H_12_O_4_	+	-	-	[51]
60	(Z)-p-coumaric acid	C_9_H_8_O_3_	+	-	-	[51]
61	p-hydroxybenzaldehyde	C_7_H_6_O_2_	+	-	-	[51]
62	p-hydroxybenzoic acid	C_7_H_6_O_3_	+	+	-	[18,51]
63	3,4-dihydroxybenzoic acid	C_7_H_6_O_4_	+	-	-	[51]
64	vanillic acid	C_8_H_8_O_4_	+	-	-	[51]
65	rosmarinic acid	C_18_H_16_O_8_	+	+	-	[18,51]
66	estra-1,3,5(10)-trien-17-*β*-ol	C_18_H_24_O	+	-	-	[55]
67	5-caffeoylquinic acid	C_16_H_17_O_9_	-	+	-	[18]
68	4-caffeoylquinic acid	C_16_H_17_O_9_	-	+	-	[18]
69	Danshensu	C_9_H_10_O_5_	-	+	-	[18]
70	(+)lyoniresinol	C_24_H_38_O_8_	-	+	-	[53]
71	(-)-5-methoxyisolariciresinol	C_22_H_30_O_7_	-	+	-	[53]
72	pinoresinol	C_21_H_26_O_6_	-	+	-	[53]
73	isoeucommin A	C_27_H_34_O_12_	-	+	-	[53]
74	episyringaresinol-4-*O*-*β*-D-glucopyranoside	C_28_H_36_O_13_	-	+	-	[53]
75	methyl-3-(3′,4′dihydroxyphenyl) lactate	C_10_H_12_O_5_	-	+	-	[56]
76	(s)-pencedanol-7-*O*-*β*-D-glucopyranoside	C_20_H_26_O_10_	-	+	-	[56]
77	stearyl ferulate	C_28_H_46_O_4_	+	-	-	[54]
Terpenoids						
(1) Monoterpene						
78	*α*-Pinene	C_10_H_16_	+	+	+	[5,26,57]
79	*β*-Pinene	C_10_H_16_	+	-	+	[5,57]
80	myrcene	C_10_H_16_	+	-	+	[5,57]
81	*β*-Phellandrene	C_10_H_16_	+	-	-	[5]
82	limonene	C_10_H_16_	+	-	+	[5,57]
83	*β*-Ocimene	C_10_H_16_	+	-	+	[5,57]
84	camphene	C_10_H_16_	+	-	+	[57,58]
85	isoterpinolene	C_10_H_16_	+	-	-	[58]
86	*γ*-terpinene	C_10_H_16_	+	+	-	[26,58]
87	4-carene	C_10_H_16_	+	-	-	[6]
88	sabinene	C_10_H_16_	+	-	+	[57,59]
89	*α*-phellandrene	C_10_H_16_	-	+	+	[26,57]
90	3-carene	C_10_H_16_	-	+	+	[26,57]
91	sylvestrene	C_10_H_16_	-	+	-	[26]
92	terpinolene	C_10_H_16_	-	+	+	[26,57]
93	*α*-thujene	C_10_H_16_	-	-	+	[57]
94	bicyclo [3.1.0]hex-2-ene, 4-methylene-1-(1-methylethyl)-	C_10_H_14_	-	-	+	[57]
95	bicyclo[4.2.0]oct-1-ene,7-exo-ethenyl-	C_10_H_14_	-	-	+	[57]
96	*α*-terpinene	C_10_H_16_	-	-	+	[57]
97	2,6-dimethyl-1,3,5,7-octatetraene	C_10_H_14_	-	-	+	[57]
98	p-mentha-1(7),2-diene	C_10_H_16_	+	-	-	[60]
99	cyclohexane, 1-methylene-4-(1-methylethenyl)-	C_10_H_16_	+	-	-	[61]
100	artemisia triene	C_10_H_16_	-	-	+	[28]
(2) Oxygenated monoterpene						
101	linalool	C_10_H_18_O	+	-	+	[5,57]
102	elsholtzia ketone	C_10_H_14_O_2_	+	-	-	[5]
103	carvone	C_10_H_14_O	+	-	-	[5]
104	dehydroelsholtzia ketone	C_10_H_12_O_2_	+	-	-	[5]
105	neodihydrocarveol	C_10_H_18_O	+	-	-	[58]
106	eucalyptol	C_10_H_18_O	+	+	-	[33,58]
107	menthone	C_10_H_18_O	+	-	-	[58]
108	linalool	C_10_H_18_O	+	-	-	[58]
109	camphor	C_10_H_16_O	+	-	-	[58]
110	eucarvone	C_10_H_14_O	+	-	-	[58]
111	perillene	C_10_H_14_O	+	-	-	[58]
112	jasmone	C_10_H_14_O	+	-	-	[58]
113	dehydroelsholtzia ketone	C_10_H_12_O_2_	+	-	-	[58]
114	eucalyptol	C_10_H_18_O	+	+	-	[33,58]
115	(−)-1R-8-hydroxy-p-menth-4-en-3-one	C_10_H_16_O_2_	+	-	-	[43]
116	fenchol	C_10_H_18_O	+	-	-	[6]
117	lavandulol	C_10_H_18_O	+	-	-	[6]
118	*α*-terpineol	C_10_H_18_O	+	-	-	[6]
119	1,6-dihydrocarveol	C_10_H_18_O	+	-	-	[6]
120	cis-carveol	C_10_H_16_O	+	-	-	[6]
121	terpinen-4-ol	C_10_H_18_O	+	+	-	[33,59]
122	nerol	C_10_H_18_O	+	-	-	[59]
123	neral	C_10_H_16_O	+	-	-	[59]
124	geraniol	C_10_H_18_O	+	-	-	[59]
125	borneol	C_10_H_18_O	-	+	-	[33]
126	trans-4-thujanol	C_10_H_18_O	-	+	-	[26]
127	sabinol	C_10_H_16_O	-	+	-	[26]
128	thymoquinone	C_10_H_12_O_2_	-	-	+	[57]
129	umbellulon	C_10_H_14_O	-	-	+	[27]
130	furan,3-methyl-2-(3-methyl-2-buten-1-yl)-	C_10_H_14_O	+	-	-	[24]
131	verbenol	C_10_H_16_O	-	-	+	[28]
132	*β*-dehydro-elsholtzione	C_10_H_12_O_2_	+	-	-	[22]
133	rose furan epoxide	C_10_H_14_O_2_	+	-	-	[65]
134	1-octen-3-yl acetate	C_10_H_18_O_2_	+	-	-	[24]
135	neryl formate	C_11_H_18_O_2_	+	-	-	[59]
136	geranyl formate	C_11_H_18_O_2_	+	-	-	[59]
137	neryl acetate	C_12_H_20_O_2_	+	-	-	[59]
138	citronellal	C_10_H_18_O	+	-	-	[6]
139	isobornyl formate	C_11_H_18_O_2_	-	-	+	[57]
(3) Sesquiterpene						
140	cubebene	C_15_H_24_	+	-	-	[5]
141	*β*-bourbonene	C_15_H_24_	+	-	-	[5]
142	*β*-caryophyllene	C_15_H_24_	+	+	+	[5,26,57]
143	*α*-caryophyllene	C_15_H_24_	+	+	-	[5,33]
144	*α*-farnesene	C_15_H_24_	+	+	+	[5,26,57]
145	*β*-bourbonene	C_15_H_24_	+	-	-	[58]
146	*β*-gurjunene	C_15_H_24_	+	-	-	[58]
147	*π*-cubebene	C_15_H_24_	+	-	-	[58]
148	*α*-bergamotene	C_15_H_28_	+	+	-	[26,58]
149	humulene	C_15_H_24_	+	-	-	[58]
150	*π*-sesquiphellandrene	C_15_H_24_	+	-	-	[58]
151	germacrene D	C_15_H_24_	+	+	-	[26,58]
152	*π*-bisabolene	C_15_H_24_	+	-	-	[58]
153	*γ*-elemene	C_15_H_24_	+	-	-	[58]
154	(Z, E)-*α*-farnesene	C_15_H_24_	+	-	-	[58]
155	caryophyllene	C_15_H_24_	+	+	-	[33,58]
156	*π*-muurolene	C_15_H_24_	+	-	-	[58]
157	*π*-cadinene	C_15_H_24_	+	-	-	[58]
158	*α*-farnesene	C_15_H_24_	+	-	-	[43]
159	ledene	C_15_H_24_	+	-	-	[43]
160	*α*-cubebene	C_15_H_24_	+	-	-	[43]
161	*γ*-cadinene	C_15_H_24_	+	-	-	[43]
162	*δ*-cadinene	C_15_H_24_	+	-	-	[43]
163	*β*-elemene	C_15_H_24_	+	-	-	[6]
164	aromadendrene	C_15_H_24_	+	-	-	[6]
165	*β*-bisabolene	C_15_H_24_	+	-	-	[59]
166	trans-*α*-bergamotene	C_15_H_24_	-	+	-	[33]
167	*γ*-muurolene	C_15_H_24_	-	+	-	[26]
168	eudesma-3,7(11)-diene	C_15_H_24_	-	+	-	[26]
169	longifolene	C_15_H_24_	-	-	+	[57]
170	trans-*α*-bergamotene	C_15_H_24_	-	-	+	[57]
171	trans-*β*-bergamotene	C_15_H_24_	-	-	+	[57]
172	(Z,E)-*α*-farnesene	C_15_H_24_	-	-	+	[57]
173	*β*-himachalene	C_15_H_24_	-	-	+	[57]
174	*γ*-cadinene	C_15_H_24_	-	-	+	[57]
175	guaiene	C_15_H_24_	-	-	+	[64]
176	*α*-zingiberene	C_15_H_24_	-	-	+	[64]
177	*α*-muurolene	C_15_H_24_	-	-	+	[64]
178	*β*-selinene	C_15_H_24_	+	-	-	[65]
(4) Oxygenated sesquiterpene						
179	(-)-humulene epoxide II	C_15_H_24_O	+	-	-	[5]
180	nerolidol	C_15_H_26_O	+	-	-	[58]
181	caryophyllene oxide	C_15_H_24_O	+	+	-	[33,58]
182	spathulenol	C_15_H_24_O	+	-	-	[6]
183	humulene-1,2-epoxide	C_15_H_24_O	-	+	-	[26]
184	cedrol	C_15_H_26_O	-	-	+	[57]
185	cedrenol	C_15_H_24_O	-	-	+	[64]
186	levomenol	C_15_H_26_O	-	-	+	[64]
187	germacrone	C_15_H_22_O	+	-	-	[22]
(5) Oxygenated diterpene						
188	2,3-dimethyl-5-(2,6,10trimethylundecyl) furan	C_20_H_36_O	+	-	-	[43]
189	phytol	C_20_H_40_O	-	-	+	[28]
(6) Triterpene						
190	forrestin A	C_30_H_42_O_11_	-	+	-	[18]
191	betulinic acid	C_30_H_48_O_3_	-	+	-	[50]
192	oleanolic acid	C_30_H_48_O_3_	-	+	-	[50]
193	ursolic acid	C_30_H_48_O_3_	-	+	-	[50]
Alkaloids						
194	N-trans-feruloyloctopamine	C_18_H_19_NO_5_	+	-	-	[51]
195	5-methyl-furan-2-carboxylic acid (1H-[1,2,4]triazol3-yl) -amide	C_8_H_8_N_4_O_2_	+	-	-	[51]
196	furane-2-carboxaldehyde, 5 (nitrophenoxymethyl)	C_12_H_9_NO_5_	+	-	-	[51]
197	uridine	C_9_H_12_N_2_O_6_	-	+	-	[18]
198	carbamult	C_12_H_17_NO_2_	-	+	-	[26]
199	phenol,O-amino-	C_6_H_7_NO	+	-	-	[61]
200	adenosin	C_10_H_13_N_5_O_4_	-	+	-	[67]
201	prunasin	C_14_H_17_NO_6_	-	+	-	[68]
202	sambunigrin	C_13_H_17_NO_7_	-	+	-	[68]
Others						
(1) Aliphatic						
203	pentacosane	C_25_H_52_	+	-	-	[55]
204	heptacosane	C_27_H_56_	+	-	-	[55]
205	octacosane	C_28_H_58_	+	-	-	[55]
206	tetratriacontane	C_34_H_70_	+	-	-	[55]
207	hexatriacontane	C_36_H_74_	+	-	-	[55]
208	n-dodecane	C_11_H_24_	-	-	+	[57]
209	n-tridecane	C_13_H_28_	-	-	+	[57]
210	n-tetradecane	C_14_H_30_	-	-	+	[57]
211	n-pentadecane	C_15_H_32_	-	-	+	[57]
212	n-hexadecane	C_16_H_34_	-	-	+	[57]
213	heptadecane	C_17_H_36_	-	+	-	[63]
214	octadecane	C_18_H_38_	-	+	-	[63]
215	tridecane,4-cyclohexyl	C_19_H_38_	-	+	-	[63]
216	nonadecane	C_19_H_40_	-	+	-	[63]
217	eicosane	C_20_H_42_	-	+	-	[63]
218	heneicosane	C_21_H_44_	-	+	-	[63]
219	docosane	C_22_H_46_	-	+	-	[63]
220	tricosane	C_23_H_48_	-	+	-	[63]
221	tetracosane	C_24_H_50_	-	+	-	[63]
222	9-cyclohexyl eicosane	C_26_H_52_	-	+	-	[63]
223	(R, R)-2,3-butanediol	C_4_H_10_O_2_	+	-	-	[58]
224	3-hexen-1-ol	C_6_H_12_O	+	-	-	[58]
225	1-hexanol	C_6_H_14_O	+	-	-	[58]
226	3-octen-1-ol	C_8_H_16_O	+	-	-	[58]
227	3-octanol	C_8_H_18_O	+	-	-	[58]
228	3,13-octadecadien-1-ol	C_18_H_34_O	-	+	-	[63]
229	2-octene-1-ol	C_8_H_16_O	+	-	-	[66]
230	hexanal	C_6_H_12_O	+	-	-	[58]
231	heptana	C_7_H_14_O	+	-	-	[58]
232	octanal	C_8_H_16_O	+	-	-	[58]
233	nonanal	C_9_H_18_O	+	-	-	[58]
234	nonacosan-10-one	C_29_H_58_O	+	-	-	[55]
235	3-hexanone	C_6_H_12_O	+	-	-	[58]
236	3-heptanone	C_7_H_14_O	+	-	-	[58]
237	3-octanone	C_8_H_16_O	+	-	-	[58]
238	irisone	C_13_H_20_O	+	-	-	[24]
239	2-pentadecanone,6,10,14-trimethyl	C_18_H_36_O	-	+	-	[63]
240	*α*-linolenic acid	C_18_H_30_O_2_	+	-	-	[9]
241	n-hexadecanoic acid	C_16_H_32_O_2_	+	-	-	[55]
242	9,12-octadecadienoic acid	C_18_H_32_O_2_	+	-	-	[55]
243	3-methylpentanoic acid	C_6_H_12_O_2_	+	-	-	[58]
244	2-methylbutanoic acid	C_5_H_10_O_2_	+	-	-	[58]
245	galactonic acid	C_6_H_12_O_7_	-	+	-	[18]
246	malic acid	C_4_H_6_O_5_	-	+	-	[18]
247	citric acid	C_6_H_8_O_7_	-	+	-	[18]
248	quinic acid	C_7_H_12_O_6_	-	+	-	[18]
249	succinic acid	C_4_H_6_O_4_	-	+	-	[18]
250	azelaic acid	C_9_H_16_O_4_	-	+	-	[18]
251	9-hydroxy-10,12-octadecadienoic acid	C_18_H_32_O_3_	-	+	-	[18]
252	palmitic acid	C_16_H_32_O_2_	-	+	-	[18]
253	oleic acid	C_18_H_34_O_2_	-	+	-	[18]
254	tetradecanoic acid	C_14_H_28_O_2_	-	+	-	[63]
255	linoleic acid	C_18_H_32_O_2_	+			[66]
256	hexadecanoic acid, ethyl ester	C_18_H_36_O_2_	+	-	-	[55]
257	9,12,15-octadecatrienoic acid methyl ester	C_19_H_32_O_2_	+	-	-	[55]
258	linoleic acid ethyl ester	C_20_H_36_O_2_	+	-	-	[55]
259	9,12,15-octadecatrienoic acidethyl ester	C_20_H_34_O_2_	+	-	-	[55]
260	octadecanoic acidethyl ester	C_20_H_40_O_2_	+	-	-	[55]
261	linolenic acide, 2-hydroxy-1(hydroxymethyl) ethyl ester	C_21_H_36_O_4_	+	-	-	[55]
262	octen-1-ol, acetate	C_10_H_18_O_2_	+	-	-	[58]
263	3-octanol, acetate	C_10_H_20_O_2_	+	-	-	[58]
264	2-propenoic acid, 2-methyl-, ethenyl ester	C_6_H_8_O_2_	+	-	-	[43]
265	diglycol laurate	C_16_H_32_O_4_	-	+	-	[18]
266	butanoic acid, 2-methylpropyl ester	C_8_H_16_O_2_	-	-	+	[57]
267	propanoic acid, 2-methyl-,butyl ester	C_8_H_16_O_2_	-	-	+	[57]
268	2-propenoic acid, 2-methyl-,butyl ester	C_8_H_14_O_2_	-	-	+	[57]
269	butanoic acid, butyl ester	C_8_H_16_O_2_	-	-	+	[57]
270	propanoic acid, 2-methyl,2-methylpropyl ester	C_8_H_16_O_2_	-	-	+	[57]
271	heptadecanoic acid, ethylester	C_19_H_38_O_2_	-	+	-	[63]
272	acetic acid, bornyl ester	C_12_H_20_O_2_	-	-	+	[34]
273	ethyl linoleate	C_20_H_36_O_2_	+	-	-	[22]
274	octenylacetate	C_10_H_18_O_2_	+	-	-	[65]
275	2,5-diethyltetrahydrofuran	C_8_H_16_O	+	-	-	[58]
276	1,8-cineole	C_10_H_18_O	+	-	-	[6]
277	dihydroartemisinin ethyl ether	C_17_H_28_O_5_	-	+	-	[18]
278	pentane, 1-butoxy-	C_9_H_20_O	-	-	+	[57]
279	ethane, 1,1-dibutoxy-	C_10_H_22_O_2_	-	-	+	[57]
280	1,1-dibutoxy-isobutane	C_12_H_26_O_2_	-	-	+	[57]
281	butane, 1,1-dibutoxy-	C_12_H_26_O_2_	-	-	+	[57]
282	3-methyl-3-oxetanemethanol	C_5_H_10_O_2_	+	-	-	[43]
283	neryl-*β*-D-glucopyranoside	C_16_H_28_O_6_	+	-	-	[51]
284	furfural	C_5_H_4_O_2_	+	-	-	[58]
285	2-acetyl-5-methylfuran	C_7_H_8_O_2_	+	-	-	[58]
286	geranyl acetate	C_12_H_20_O_2_	+	-	-	[58]
287	octen-3-ol	C_9_H_19_O	+	-	+	[6,57]
288	cis-jasmone	C_11_H_16_	+	-	-	[6]
289	2,6-octadien-1-ol, 3,7-dimethyl-	C_11_H_20_	-	-	+	[57]
290	3,5-dimethylcyclohex-1-ene-4carboxaldehyde	C_9_H_14_O	-	+	-	[33]
291	(6S,9R)-roseoside	C_19_H_30_O_8_	-	+	-	[67]
292	1-hexadecene	C_16_H_32_	-	+	-	[63]
293	1-nonene	C_9_H_18_	-	-	+	[34]
294	ligustilide	C_12_H_14_O_2_	+	-	-	[22]
295	cyclohexene, 2-ethenyl-1,3,3-trimethyl	C_11_H_18_	+	-	-	[43]
296	daucosterol	C_35_H_60_O_6_	+	-	-	[54]
(2) Aromatic						
297	thymol	C_10_H_14_O	+	+	+	[33,57,58]
298	carvacrol	C_10_H_14_O	-	+	+	[33,57]
299	p-cymene	C_10_H_14_	+	+	+	[6,26,57]
300	methyl chavicol	C_10_H_12_O	+	-	-	[59]
301	m-cymene	C_10_H_14_	-	-	+	[57]
302	3,4-diethylphenol	C_10_H_14_O	-	-	+	[57]
303	p-eugenol	C_10_H_12_O_2_	-	-	+	[57]
304	O-cymene	C_10_H_14_	+	-	-	[60]
305	cis-anethol	C_10_H_12_O	-	+	-	[62]
306	3,4,5-trimethoxytoluene	C_10_H_14_O_3_	-	+	-	[62]
307	cis-asarone	C_10_H_14_O_2_	-	+	-	[63]
308	cuminaldehyde	C_10_H_12_O	-	-	+	[64]
309	benzyl-*β*-D-glucopyranoside	C_13_H_18_O_6_	+	-	-	[51]
310	p-xylene	C_8_H_10_	+	-	-	[58]
311	benzaldehyde	C_7_H_6_O	+	-	+	[57,58]
312	thymol acetate	C_12_H_16_O_2_	+	+	+	[33,57,58]
313	acetophenone	C_8_H_8_O	+	-	-	[58]
314	naphthalene	C_10_H_8_O	+	-	-	[43]
315	protocatechuic acid	C_7_H_6_O_4_	-	+	-	[18]
316	sodium ferulate	C_10_H_9_NaO_4_	-	+	-	[18]
317	4,5-dimethyl-2-ethylphenol	C_10_H_14_O	-	+	-	[33]
318	thymyl acetate	C_12_H_16_O_2_	-	+	-	[26]
319	carvacryl acetate	C_12_H_16_O_2_	-	+	-	[26]
320	4-*O*-*β*-D- glucopyranosylbenzyl-4′-hydroxylbenzoate	C_20_H_22_O_9_	-	-	+	[39]
321	4-*O*-*β*-D-glucopyranosylbenzyl-3′-hydroxy-4′- methoxybenzoate	C_21_H_24_O_10_	-	-	+	[39]
322	amburoside A	C_20_H_22_O_10_	-	-	+	[39]
323	4-[[(2′,5′-Dihydroxybenzoyl)oxy]methyl]phenyl-*O*-*β*-D-glucopyranoside	C_20_H_20_O_10_	-	-	+	[39]
324	hyprhombin B methyl ester	C_26_H_22_O_9_	-	-	+	[39]
325	methyl lithospermate	C_28_H_24_O_12_	-	-	+	[39]
326	dimethyl lithospermate	C_29_H_26_O_12_	-	-	+	[39]
327	salvianolic acid C methyl ester	C_27_H_23_O_10_	-	-	+	[39]
328	sebestenoids C	C_36_H_30_O_14_	-	-	+	[39]
329	cucurbitoside D	C_25_H_30_O_13_	-	-	+	[39]
330	2,4-di-tert-butylphenol	C_14_H_22_O	-	-	+	[27]
331	benzyl benzoate	C_14_H_12_O_2_	+	-	-	[69]
332	4-isopropyl-3-methylphenol	C_10_H_14_O	+	-	-	[60]
333	benzenemethanol, 4-(1-methylethyl)*-*	C_10_H_14_O	+	-	-	[61]
334	methyl eugenol	C_11_H_14_O_2_	-	+	-	[62]
335	elemicin	C_12_H_16_O_3_	-	+	-	[62]
336	myristicin	C_11_H_12_O_3_	-	+	-	[62]
337	methyl thymol ether	C_11_H_16_O	-	-	+	[28]
338	apiole	C_12_H_14_O_4_	-	-	+	[28]
339	4-hydroxy-2,6-dimethoxyphenyl-*β*-D-glucopyranoside	C_14_H_20_O_9_	-	+	-	[67]
340	4-hydroxy-3,5-dimethoxyphenyl-*β*-D-glucopyranoside	C_14_H_20_O_9_	-	+	-	[67]
341	3,4,5-dimethoxyphenyl-*β*-D-glucopyranoside	C_15_H_22_O_9_	-	+	-	[67]
342	3-hydroxyestragole-*β*-D-glucopyranosides	C_16_H_22_O_7_	-	+	-	[67]
343	4-acetoxy-3-methoxy acetophenone	C_11_H_12_O_4_	-	+	-	[63]
344	benzyl-D-glucopyranoside	C_13_H_18_O_6_	-	+	-	[63]
345	2′,4′-dihydroxy-3′methylacetophenone	C_9_H_10_O_3_	-	-	+	[34]
346	acetylthymol	C_12_H_16_O_2_	-	-	+	[64]
347	carvacrol acetate	C_12_H_16_O_2_	+	-	-	[66]
348	bergaptene	C_12_H_8_O_4_	+	-	-	[66]
349	2-butenylbenzene	C_10_H_12_	+	-	-	[22]
350	*α*-methyl-benzenepropanol	C_10_H_14_O	+	-	-	[22]
351	cuminic acid	C_10_H_12_O	+	-	-	[22]
352	p-cymen-8-ol	C_11_H_16_O	+	-	-	[6]

“+” Found in plants. “-” Not found in plants.

## 4. Conclusions and Prospects

This paper summarizes pharmacological activities of *E. ciliata*, among which antioxidant, anti-inflammatory, antimicrobial and insecticidal activities are the main activities, but also has antiviral, hypolipidemic, hypoglycemic, anti-tumor activities. Hence, **352** kinds of chemical constituents identified from *E. ciliata* were summarized. According to their structure types, they can be divided into flavonoids, phenylpropanoids, terpenoids, alkaloids and other compounds.

According to the existing pharmacological experiment results in vivo and in vitro, *E. ciliata* dichloromethane extract, ethyl acetate extract and essential oil all show good pharmacological activity. Carvacrol contained in *E. ciliata* is the main active ingredient of antibacterial. At present, researches on pharmacological activity of *E. ciliata* mainly focus on essential oil, and some researches involve *E. ciliata* alcohol extract, water extract and polysaccharide, but there are relatively few researches on pharmacological activities of *E. ciliata* such as analgesia, immune regulation, hypoglycemia and hypolipemia. Whether *E. ciliata* has potential pharmacological activity still needs further test to prove. The safety study of clinical dose also deserves extensive attention. 

**Figure 2:**
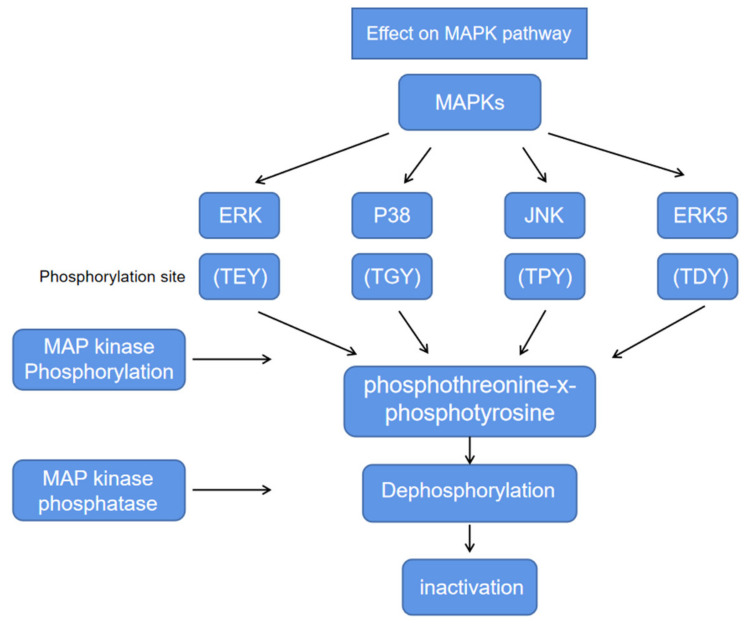
Effect on Mitogen-activated protein kinases pathway.

**Figure 3:**
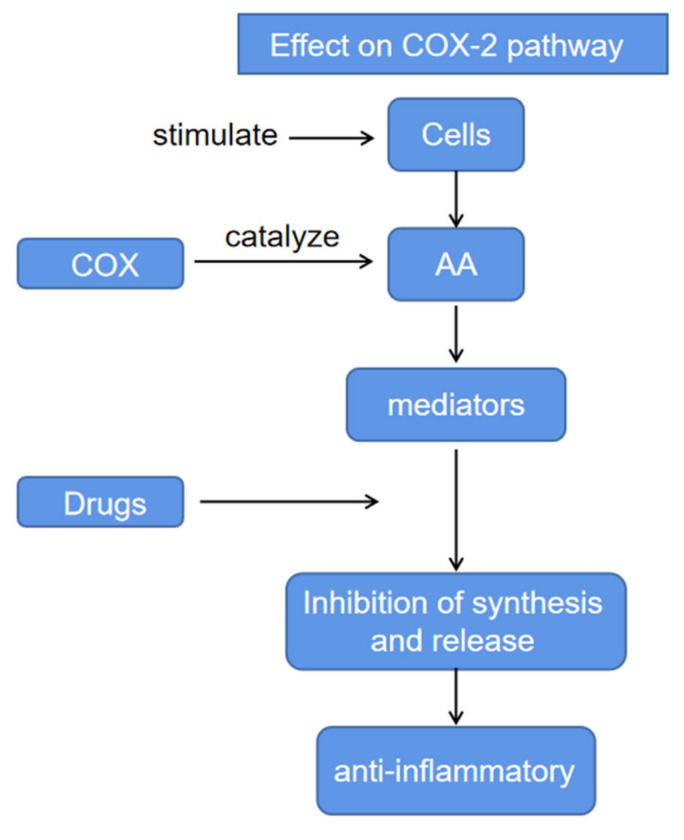
Effect on Cyclooxygenase-2 pathway.

Some representative action mechanisms of *E. ciliata* were briefly illustrated for the sake of reference. The possible action process is shown in the figure. The mitogen-activated protein kinases (MAPKs) signaling pathway chain consists of three protein kinases, MAP3K–MAP2K–MAPK, which transmit upstream signals to downstream responsive molecules through sequential phosphorylation. MAPK includes four subfamilies: ERK, p38, JNK, and ERK5. MAPK activity is thought to be regulated by the diphosphate sites in the amino acid sequence of the active ring. The active ring contains a characteristic threonine-x-tyrosine (T-x-Y) motif. Mitogen activated protein (MAP) kinase phosphorylates on two amino acid residues, thereby activating MAPK pathway. MAP kinase phosphatase (MKP) can hydrolyze phosphorylated products and inactivate MAPK pathway. The extract inhibited the activation of MAPK signaling pathway by blocking the phosphorylation of p38, JNK and ERK [18]. When stimulated, tissue cells release arachidonic acid (AA). Cyclooxygenase (COX) catalyze AA to produce a series of bioactive substances such as prostaglandins (PGs), causing inflammation. The extract can affect the COX-2 pathway by affecting the release levels of TNF-*α*, IL-6, and PGE2, which are key mediators released by macrophages during bacterial infection, so as to achieve the purpose of anti-inflammatory [4]. Carvacrol can significantly inhibit the mRNA expression of toll like receptor 7 (TLR7), interleukin-1 receptor associated kinase (IRAK4), TNF receptor associated factor (TRAF6), induced pluripotent stem-I (IPS-I), and interferon regulatory factor 3 (IRF3) in mice, thereby affecting the immunomodulatory signaling pathways of TLR7/RLR and playing an anti-H1N1 influenza virus role [19]. With the deepening of various studies, the gradual clarification of the mechanism of action has created conditions for drugs to play a better role. Two representative mechanisms of action, MAPK and COX-2, are shown in Figure 2 and Figure 3.

*E. ciliata* has rich resources and low requirements for growth environment. It can be planted artificially and has a short growth cycle. The rich essential oil content makes it possible for *E. ciliata* to be used as flavor and food additive. Undoubtedly, the development of *E. ciliata* in new dosage forms and the application to medicine, food, and other fields will provide broad development prospects in the future.

## Data Availability

The data presented in this study are available on request from the corresponding author.
